# 肺癌气腔扩散的研究进展

**DOI:** 10.3779/j.issn.1009-3419.2021.101.49

**Published:** 2022-01-20

**Authors:** 蕾 范, 萍 何

**Affiliations:** 510120 广州，广州医科大学附属第一医院病理科 Department of Pathology, First Affiliated Hospital of Guangzhou Medical University, Guangzhou 510120, China

**Keywords:** 肺腺癌, 气腔扩散, 预后, Lung adenocarcinoma, Spread through the air spaces, Prognosis

## Abstract

气腔扩散（spread through air spaces, STAS）是2015版世界卫生组织（World Health Organization, WHO）肺癌病理分类中首次提出的概念，是指在主瘤体边界外的气腔内出现微乳头状细胞簇、实性细胞巢或单个肿瘤细胞。除传统观念的肺腺癌浸润方式（间质、脉管及胸膜侵犯）外，STAS被确定为第四种肺浸润性腺癌的侵袭模式。近年来，关于STAS的研究成为热点，STAS的存在不仅与肺癌病理组织学、基因突变等因素相关，诸多研究也证实其可作为肿瘤复发及预后的独立相关因素。但是，也有研究认为人为因素可以造成STAS的形态学假象，需在临床工作中注意甄别。本文就STAS的分类、相关病理学特征、基因状态改变以及可能造成STAS假象的人为因素等方面的研究进展予以综述。

## Abstract (en)

The concept of spread through air spaces (STAS) was first proposed in the World Health Organization (WHO) Classification of Tumors of the Lung, Pleura, Thymus and Heart (version 2015). STAS is defined as the micropapillary clusters, solid nests or single cells of tumor that exist in the air spaces of the surrounding lung parenchyma beyond the edge of the main tumor. Meanwhile, apart from the traditional invasion modes of lung adenocarcinoma (interstitial, visceral pleura and lym-phovascular invasion), STAS has been identified as the fourth invasion mode of lung adenocarcinoma. In recent years, the research on STAS has been a hot spot in the field of lung adenocarcinoma. The existence of STAS is related to lung cancer histopathology, gene mutation and other factors, and many studies have also confirmed that it can be used as an independent factor for tumor recurrence and prognosis. However, according to some studies, human factors can cause morphological artifacts of STAS, which still needs to be distinguished in clinical work. This paper reviews the research progress of STAS classification, related pathological features, genetic status changes, and human factors that may cause STAS artifacts.

近年来，随着医学影像技术的发展，早期肺癌的检出率及治愈率在逐步增高，但2020年全球癌症数据统计显示，2020年约有180万人因肺癌死亡，肺癌仍然是癌症死亡的主要原因^[1]^，其中肺腺癌为最常见的组织学亚型。早期肺癌通过手术治疗一般可获得较好的预后，生存率及治愈率明显提高，但随着肺癌转移方式——气腔扩散（spread through air spaces, STAS）概念的提出，研究者们发现STAS可能是肺癌不良预后的独立影响因子。自2015版世界卫生组织（World Health Organization, WHO）肺癌病理分类中首次提出STAS的概念后，众多研究者对STAS与肿瘤主体生长方式之间的关系、肺癌患者的病理组织学特征、基因状态改变及患者预后等方面进行了一定的研究，结果表明STAS在早期及中晚期肺癌中均与以上诸多因素具有显著相关性，STAS可成为肺癌不良预后的独立因素之一。STAS的病理诊断对患者手术方式的选择及预后判断可能具有一定的指导意义。目前STAS已成为肺癌研究领域的热点之一。本文就STAS的分级、相关病理学特征、基因状态改变、STAS对预后及治疗方式的影响以及可能造成STAS假象的人为因素等方面的研究进展予以综述。

## 1 STAS的分级

2013年，Onozato等^[2]^通过三维重建的方法在肺腺癌的标本中发现“孤立于肺泡腔内的肿瘤细胞”，并首次将其定义为“肿瘤岛”。研究者对“肿瘤岛”与肺癌预后的关系进行了分析，文章指出存在“肿瘤岛”现象的病例复发率较未出现“肿瘤岛”的病例高。2015年，WHO正式提出STAS的概念，并对其进行了形态学定义，但并未给出STAS的量化标准。随后有学者对STAS进行了不同分类。Warth等^[3]^研究了569例肺浸润性腺癌，每个病例至少具有一张包含肿瘤完整边界与相邻肺组织的病理切片，根据STAS细胞簇与主肿瘤之间的距离进行分级，以3个肺泡为界，将距离小于3个肺泡者定义为局限性STAS，将距离大于3个肺泡者定义为广泛性STAS，文章发现广泛性STAS更易出现于以高级别生长方式为主的肺腺癌。Toyokawa等^[4]^对276例Ⅰ期肺腺癌患者进行STAS回顾性整理，根据肿瘤细胞的数量将STAS分为三类：“no STAS”（未见明确肿瘤细胞）、“low STAS”（1个-4个单个或簇状肿瘤细胞）及“high STAS”（5个及5个以上单个或簇状肿瘤细胞）。文章指出，STAS的细胞数量与肿瘤大小、胸膜侵犯及周围组织侵犯等具有统计学相关性，具有STAS的肿瘤预后相对更差。Han等^[5]^将未进行新辅助治疗且无其他肿瘤史的1, 869例非小细胞肺癌病例进行汇总，研究采用两级分级系统，根据STAS细胞簇与主肿瘤之间的距离分级，将距离小于2, 500 μm[一个×10物镜的视野]定义为STAS Ⅰ级，距离大于2, 500 μm[一个×10物镜的视野]定义为STAS Ⅱ级，文章比较了无复发生存率、总生存期、肺癌特异性生存率等诸多方面，STAS Ⅰ级预后均优于STAS Ⅱ级。综上所述，STAS的扩散距离或肿瘤细胞数量对肿瘤发展及预后具有一定的影响，所以STAS量化标准的统一有助于对肺腺癌预后的精准判断及治疗方案的合理选择。

## 2 STAS的相关病理学特征

2015年，Kadota等^[6]^提出STAS几种组织学亚型：①微乳头结构，由没有纤维血管轴心的乳头结构组成，偶尔在空气中形成环状结构；②实体巢，由实体肿瘤细胞组成；③单个细胞，由散在的松散的单个细胞组成（图 1）。根据2021版WHO肺癌病理分类，肺腺癌肿瘤高级别生长方式包含以下几种：微乳头状、实体状、筛孔状及复杂腺体结构等。研究表明以高级别生长方式为主的肿瘤中更易出现STAS，且多以广泛性STAS为主^[3-6]^。Terada等^[7]^筛选出76例接受肺叶或全肺切除术且已完成系统性淋巴结剥离的肺腺癌患者进行分析，其中46例为STAS阳性，发现STAS阳性率与肿瘤生长方式中的乳头状成分及微乳头状成分具有统计学相关性。Sun等^[8]^以微乳头状生长模式肺腺癌为主要研究对象，其中有微乳头状和无微乳头状生长方式肿瘤的STAS阳性率分别为80.9%和55.9%，二者差别具有统计学意义，表明STAS阳性率与肺癌生长模式具有相关性。Alvarez等^[9]^在240例肺癌患者中发现67例为STAS阳性，其中STAS在以微乳头状生长方式为主的肺腺癌中的发生率约占70.0%，明显高于其他肿瘤生长模式。Lee等^[10]^在316例肺腺癌患者中发现STAS阳性者达160例（50.5%），且常见于筛状（占10%）、微乳头状（占14.4%）和实体状生长方式（占22.5%）为主的肿瘤。Xie等^[11]^也在文章中证实这一观点，STAS与高级别肺腺癌成分如筛状、微乳头状或实体状生长方式密切相关。

**图 1:**
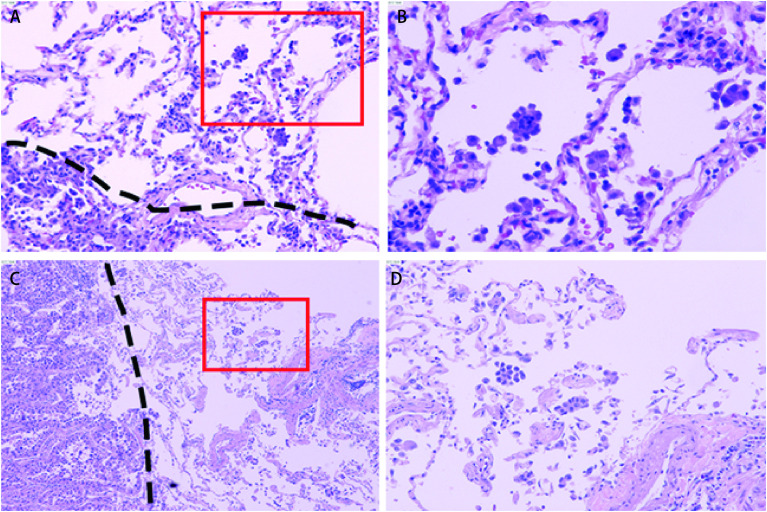
STAS的形态学特征（HE，染色A、D×100；B×200；C×40）。A：浸润性腺癌（虚线以下为肿瘤主体）；虚线：肿瘤边界；红色方框：STAS；B：STAS（微乳头状及单细胞状）；C：浸润性腺癌（虚线以左为肿瘤主体）；虚线：肿瘤边界；红色方框：STAS；D：STAS（小巢状及单细胞状）。 Morphologic features of spread through air spaces (STAS) pattern (original magnification : ×100 in A and D; ×200 in B; ×40 in C). A: Invasive adenocarcinoma (The primary tumor is below the dotted line); Dotted line: Tumor boundary; Red box: STAS; B: STAS: Micropapillary clusters and single cells; (C) Invasive adenocarcinoma (The primary tumor is to the left of the dotted line); Dotted line: Tumor boundary; Red box: STAS; (D) STAS: Small solid nests and single cells.

此外，有研究^[6, 12]^表明STAS与侵袭性临床病理特征显著相关，例如肿瘤分期、分化程度、肿瘤大小、胸膜浸润及脉管侵犯等。Tian等^[13]^统计分析了已完成系统淋巴结清扫且已被病理证实为STAS阳性的878例肺癌患者，其中包括813例（92.6%）肺腺癌患者和25例（2.8%）肺鳞癌患者，研究者发现肿瘤体积越大，STAS阳性率越高。Sun等^[8]^根据肿瘤大小对肺腺癌患者进行分组分析，结果显示STAS阳性率在肿瘤 > 3 cm的病例中明显高于肿瘤≤3 cm的病例。Sun等^[8]^根据病理特征将STAS阳性患者进行分组，其中胸膜侵犯、脉管侵犯组STAS阳性率明显高于神经束侵犯及肿瘤性坏死组。Han等^[5]^发现在肺腺癌中具有胸膜侵犯、淋巴血管侵犯和（或）病理分期较高等特征的患者更易出现STAS。

目前对于STAS的研究多集中在肺腺癌，但有研究显示，其他组织学类型的肺癌中同样存在STAS现象。2016年，Lu等^[14]^在肺鳞癌中发现STAS现象，并指出STAS出现概率与肿瘤分期呈正相关。STAS阳性患者的复发率和死亡率均高于STAS阴性的患者，STAS是肺鳞状细胞癌具有预后意义的相关因素之一。

## 3 STAS的相关基因突变

目前已有较多关于STAS现象与肺癌基因状态的相关研究（表 1）。多数研究认为STAS与野生型表皮生长因子受体（epidermal growth factor receptor, EGFR）基因状态相关。Onozato等^[2]^提出“肿瘤岛”概念时，同时指出具有肿瘤岛的肺腺癌多未发现*EGFR*突变，而以贴壁式生长为主的腺癌更易出现*EGFR*基因突变。Lee等^[10]^通过*Logistic*回归分析显示，没有贴壁状生长方式、以微乳头状及筛孔状生长方式占主导、具有脉管侵犯的STAS阳性的肿瘤与野生型EGFR显著相关。多篇文献均证实STAS与野生型EGFR基因状态相关^[3, 8, 12, 15]^。

**表 1:** STAS的出现频率及相关病理学特征 STAS frequency and pathological features

Authors	Patients (*n*)	Primary diagnosis	AJCC stage	STAS (%)	Histology	Mutations
onozato 2013^[2]^	261	ADC	Ⅰ-Ⅱ	22.2%	Solid or micropapillary pattern	*KRAS*(+)
Warth 2015^[3]^	569	ADC	Ⅰ-Ⅳ	50.6%	Micropapillary, solid or cribriform pattern	*KRAS*(-), *BRAF*(+), *EGFR*(+)
Han 2021^[5]^	1869	NSCLC	Ⅰ-Ⅳ	40.9%	Micropapillary and solid pattern	*EGFR*(-)
Kadota 2015^[6]^	411	ADC	NR	38.0%	Micropapillary and solid pattern	NR
Terada 2019^[7]^	76	ADC	Ⅲ(N2)	60.5%	Micropapillary and papillary pattern	NR
Sun 2017^[8]^	288	ADC	Ⅰ-Ⅳ	61.8%	Micropapillary pattern	*KRAS*(-), *EGFR*(+), *ALK*(+), *ROS1*(+)
Alvarez 2021^[9]^	240	Lung cancer	NR	27.9%	Micropapillary pattern	NR
Lee 2018^[10]^	316	ADC	NR	50.6%	Micropapillary, cribriform or solid pattern	*KRAS*(-)、*EGFR*(-)、*ALK*(+)、*ROS1*(+)
Xie 2021^[11]^	803	NSCLC	Ⅰ-Ⅳ	46.1%	Micropapillary or solid pattern	NR
Hu 2018^[12]^	500	ADC	Ⅰ-Ⅲ	26.8%	Micropapillary or solid pattern	*KRAS*(-), *BRAF*(-), *EGFR*(-), *ALK*(+), *Her-2*(-)
Tian 2021^[13]^	878	Lung cancer	Ⅰ-Ⅳ	100.0%	Micropapillary or solid pattern	*KRAS*(+), *EGFR*(+), *Her-2*(+)
Lu 2017^[14]^	445	SCC	Ⅰ-Ⅲ	30.0%	NR	NR
Shiono 2016^[15]^	318	ADC	Ⅰ	14.8%	NR	*EGFR*(-)
Jia 2020^[16]^	303	ADC	Ⅰ-Ⅳ	60.4%	Micropapillary pattern	*EGFR*(-), *ALK*(+), *ROS-1*(+)
	121	SQCC	NR	32.2%	NR	
ADC: adenocarcinoma; NSCLC: non-small cell lung cancer; SCC: squamous cell carcinoma; SQCC: squamous cell carcinoma; STAS: spread through air spaces; AJCC: American Joint Committee on Cancer; EGFR: epidermal growth factor receptor; ALK: anaplastic lymphoma kinase.						

目前在STAS分子基因研究领域，不同研究者的结论不完全一致。Onozato等^[2]^提出*KRAS*基因突变的患者中有46%出现肿瘤岛，证实肿瘤岛的出现与*KRAS*基因突变具有统计学相关性。Tian等^[13]^在STAS分子基因研究中，发现KRAS基因突变的患者占比12%，文章指出STAS与*KRAS*基因突变具有统计学相关性，且在男性中更为普遍。但Jia等^[16]^通过对161例STAS病例进行分子研究，其中91.9% *KRAS*基因突变为阴性，提示二者无相关性。Lee等^[10]^的研究结果也表明STAS与*KRAS*基因突变无相关性。此外，还有多位学者^[8, 10]^在研究中提出STAS在肺腺癌中与*ALK*和*ROS1*基因重排相关，STAS多见于*ALK*重排的肺腺癌^[10]^。Jia等^[16]^对肺腺癌及肺鳞癌的STAS现象进行了综合分析，研究者发现在183例STAS阳性的肺腺癌中，STAS与*ALK*和*ROS1*重排、低E-钙粘蛋白表达以及高波形蛋白和Ki67表达相关；在39例STAS阳性的肺鳞癌中，STAS与低E-钙粘蛋白表达和高波形蛋白和存活蛋白表达相关。

Tian等^[13]^对STAS阳性的139例肺腺癌病例进行分子基因检测，结果表明在肿瘤≤3 cm的病例中，*EGFR*基因突变、*TP53*基因突变、*KRAS*基因突变、*ALK*和*ROS1*基因重排是STAS阳性肺腺癌中最常见的5种改变。也有相关研究^[12]^表明STAS阳性的患者更多地表现为*EGFR*（-）、*KRAS*（-）、*BRAF*（-）、*ALK*（+）和*HER2*野生型。

## 4 STAS对预后及治疗方式的影响

2019年，Chen等^[17]^将非小细胞肺癌按组织学类型分组分析，在肺腺癌、肺鳞癌及肺多形性癌中，STAS与术后无复发生存率显著相关。随着Chen等^[17]^研究数据的持续增加，STAS的预后价值更为明显，与Ⅳ期非小细胞肺癌的预后存在明确相关性，同时在局部切除手术中，无复发生存率较STAS阴性者明显下降。但在接受肺叶切除术的非小细胞肺癌中，STAS是否影响无复发生存率仍存在疑问。STAS是肺腺癌的独立预后不良因素^[15, 18]^，STAS阳性的患者无进展生存期与总生存期均较短^[8]^。在Han等^[5]^学者提出的两级分级系统中，早期肺腺癌阶段，STAS Ⅱ级（肿瘤细胞巢与主肿瘤距离大于2, 500 μm）与较短的无复发生存率和肺癌症状评分显著相关，在接受局部切除或肺叶切除术患者中均是独立的不良预后因素。但在Han等^[5]^研究发现STAS Ⅰ级在多变量分析中与复发无关。Chen等^[17]^也提出在肿瘤边缘出现的STAS目前对于预后的影响暂不确定。所以STAS的分级对肿瘤的预后存在一定的影响。Xie等^[11]^通过对STAS的生长模式进行分析，发现微乳头状或实体巢状型STAS为预后不良的独立因素，而单细胞型STAS被认为是无预后意义的STAS生长模式。

肺腺癌的手术方式分为肺段或楔形肺切除（局部切除）以及肺叶切除方式。研究^[5]^发现STAS Ⅱ级是局部切除组和肺叶切除组肿瘤复发的独立不良预后因素。有研究^[19]^结果表明STAS在局部切除组中的复发率明显高于肺叶切除组。局部切除组中，STAS阳性患者的复发风险显著高于阴性患者^[6, 19]^，并且5年无病生存率明显低于阴性患者^[19]^。在多变量分析中，肿瘤STAS的存在是任何复发的独立且唯一的危险因素^[6]^，当存在STAS时，肿瘤局部复发和远处复发的风险均较高^[20]^。Onozato等^[21]^根据模型预测显示STAS阳性与阴性病例在肺叶切除组中总生存率无显著差异，但在非肺叶切除组中具有显著差异。所以术中冰冻诊断STAS可对术式选择提供参考，但目前由于冰冻取材的限制，STAS的诊断具有一定局限性，因此提高术中STAS检出率，对于合理术式选择尤为重要。

许多研究已证实STAS对肺腺癌预后的重要影响，少有研究评估STAS在鳞状细胞癌^[14, 22]^和神经内分泌肿瘤中的作用^[23, 24]^。Travis等^[25]^提出STAS可能在肺神经内分泌癌或肺类癌中的预后相对较好，STAS对不同组织学类型肺癌的预后影响程度不同，非腺癌类型方面还需更多研究数据支持。

## 5 可能导致STAS假象的人为因素

STAS概念自提出以来，诸多回顾性研究均表明STAS现象具有独特生物学意义，但因不能完全排除STAS是人为因素造成的形态学假象，所以该概念仍存在争议^[26]^。有学者对这些假阳性现象的镜下特点进行了研究，Xie等^[11]^在研究过程中发现多数假阳性现象为单细胞型STAS。肺泡腔内出现的单个细胞应与巨噬细胞相鉴别^[15]^，镜下所见缺乏持续扩散过程且随机漂浮于肺泡腔内的单个肿瘤细胞、从肺泡壁脱落的线性细胞带及锯齿状细胞团簇应提示人为假象^[14]^（图 2）。此外，位于组织边缘或与已知肿瘤主体组织学类型不一致的肿瘤细胞，多考虑人为假象。现有对STAS人为假象进行研究的文献中提出，取材过程中，若刀面上黏附少许肿瘤碎片或细胞簇^[27, 28]^，或因术中肿瘤组织受到人为挤压，从而导致肿瘤细胞脱落至肺泡腔内^[28]^，均可造成STAS假阳性现象。

**图 2:**
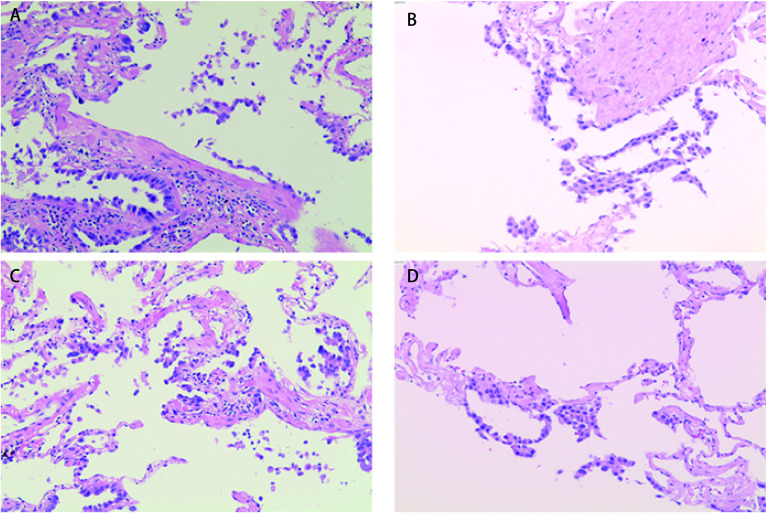
人为因素导致的STAS假象（HE，染色×100）。A：肺泡壁脱落的线性细胞带；B：肺泡壁脱落的线性细胞带；C：锯齿状细胞团簇；D：肺泡壁脱落的线性细胞带。 Morphologic artifacts of the pattern of tumor STAS caused by human factors (HE staining, ×100). A: Linear strips of cells lifted off alveolar walls; B: Linear strips of cells lifted off alveolar walls; C: Clusters of serrated cells; D: Linear strips of cells lifted off alveolar walls.

Lu等^[14]^在研究中发现2例没有切除主肿瘤，却仍然出现STAS的病例，因此认为STAS并不是单纯的因手术切除或者外力挤压等造成的现象，而是一种生物学现象，是一种侵袭方式。但研究者并未对如何鉴别人为STAS及真正的STAS的方法进行深入探讨。因此，对STAS进行标准量化研究首先要注意鉴别人为因素导致的STAS形态学假象^[29]^。

为避免上述几种造成STAS人为假象的可能，需在工作中注意以下几点：首先，处理标本阶段应遵循标准化流程，避免组织污染；应对主肿瘤周围组织充分取材，确保对STAS判断的准确性^[30]^。其次，组织病理镜下分析阶段，应鉴别吞噬细胞与单个肿瘤细胞型STAS，必要时可运用免疫组化进行鉴别。但术中冰冻病理因取材受限，对于鉴别STAS与人为假象仍存在一定困难。

## 6 小结

STAS作为一种新的侵袭方式，已被证实在肺腺癌、鳞癌及多形性癌中均与脉管侵犯、胸膜侵犯等病理学特征有关，是肺腺癌的独立预后不良因素。但在肺癌不同组织学亚型或不同手术方式中，STAS对预后的影响也有所区别。STAS的分析对于肺癌的病理评估十分重要，目前对于STAS现象与人为假象的鉴别尚存在一定困难，尤其是在术中冰冻病理诊断中。因多种因素受限，术中无法快速鉴别STAS与人为假象，可能影响手术方式的选择，对于STAS阳性的患者如果仅仅进行局限性切除，有可能会导致不良预后。此外，一般而言肿瘤细胞需依附血管而存在，STAS作为漂浮于肺泡腔内的单个细胞或细胞簇，其具体形成机制及肿瘤细胞存活机制暂不明确，所以STAS的发生机制仍需进一步探究。
